# Overexpression of *ZmIRT1* and *ZmZIP3* Enhances Iron and Zinc Accumulation in Transgenic *Arabidopsis*

**DOI:** 10.1371/journal.pone.0136647

**Published:** 2015-08-28

**Authors:** Suzhen Li, Xiaojin Zhou, Hongbo Li, Yuanfeng Liu, Liying Zhu, Jinjie Guo, Xiaoqing Liu, Yunliu Fan, Jingtang Chen, Rumei Chen

**Affiliations:** 1 Department of Agronomy, Agricultural University of Hebei/Hebei Sub-center of Chinese National Maize Improvement Center, Baoding, China; 2 Department of Crop Genomics & Genetic Improvement, Biotechnology Research Institute, Chinese Academy of Agricultural Sciences, Beijing, China; Institute of Genetics and Developmental Biology, Chinese Academy of Sciences, CHINA

## Abstract

Iron and zinc are important micronutrients for both the growth and nutrient availability of crop plants, and their absorption is tightly controlled by a metal uptake system. Zinc-regulated transporters, iron-regulated transporter-like proteins (ZIP), is considered an essential metal transporter for the acquisition of Fe and Zn in graminaceous plants. Several ZIPs have been identified in maize, although their physiological function remains unclear. In this report, *ZmIRT1* was shown to be specifically expressed in silk and embryo, whereas *ZmZIP3* was a leaf-specific gene. Both ZmIRT1 and ZmZIP3 were shown to be localized to the plasma membrane and endoplasmic reticulum. In addition, transgenic *Arabidopsis* plants overexpressing *ZmIRT1* or *ZmZIP3* were generated, and the metal contents in various tissues of transgenic and wild-type plants were examined based on ICP-OES and Zinpyr-1 staining. The Fe and Zn concentration increased in roots and seeds of *ZmIRT1*-overexpressing plants, while the Fe content in shoots decreased. Overexpressing *ZmZIP3* enhanced Zn accumulation in the roots of transgenic plants, while that in shoots was repressed. In addition, the transgenic plants showed altered tolerance to various Fe and Zn conditions compared with wild-type plants. Furthermore, the genes associated with metal uptake were stimulated in *ZmIRT1* transgenic plants, while those involved in intra- and inter- cellular translocation were suppressed. In conclusion, ZmIRT1 and ZmZIP3 are functional metal transporters with different ion selectivities. Ectopic overexpression of *ZmIRT1* may stimulate endogenous Fe uptake mechanisms, which may facilitate metal uptake and homeostasis. Our results increase our understanding of the functions of ZIP family transporters in maize.

## Introduction

Micronutrients are essential for both plant growth and human health, and a deficiency in such nutrients may reduce the yield and quality of crop plants and lead to nutritional deficiency syndrome in mammals such as anaemia, hypophrenia, and stagnation of growth and development. It was reported that deficiencies in bio-available iron, zinc, and other essential minerals affect a large proportion of the global population [1–3]. Besides, zinc and iron are essential metal nutrient factors for plants since they play critical roles in the process of growth and development, including photosynthesis, respiration, and other biochemical reactions that require Zn or Fe as co-factors.

In plants, zinc deficiency leads to internode shortening, reduction in leaf size and other morphological changes [4–6], while iron deficiency can cause severe chlorosis and growth arrest. A deficiency in Zn and Fe repress plant growth and reduces the yield and quality [7], although too much Zn and Fe may result in considerable biochemical toxicity [8, 9]. To avoid excessive absorption and facilitate adequate intake, plants have created a balanced network to regulate the uptake, utilisation, and storage of these metal ions [10, 11]. Actually, such adjustments rely on genes that control ion homeostasis in plants. Recently, several metal transporters that contribute to metal-ion homeostasis in plants have been identified, including the zinc-regulated transporter (ZRT), iron-regulated transporter (IRT)-like protein (ZIP), natural resistance-associated macrophage protein (NRAMP), and yellow stripe-like protein (YSL).

Zn exhibits low solubility, and its solubilisation is thought to occur via acidification of rhizosphere and secretion of organic chelators such as nicotianamine (NA) and citric acid. Subsequently, as a free ion, zinc is uptake into the root cells by ZIPs [12]. Zn can then be transported into vacuoles and immobilised in the root symplast or it can be translocated into the vascular cylinder through plasmodesmata [12]. The translocation of Zn from roots to shoots requires the export of zinc from root cells and loading of zinc into the apoplastic xylem [13, 14]. Inside the xylem, zinc flux from roots to shoots is massflow driven and ionic zinc is chelated by low-molecular-weight ligands to avoid Zn retention. Then, Zn is taken up from the xylem of shoots and transported across the plasma membranes of adjacent cells via membrane zinc transporters [15]. Two different mechanisms are involved in iron acquisition in plants. Strategy I is specifically used by nongraminaceous plants, which contains the reduction of Fe^3+^ to Fe^2+^ on the root surface and the acquisition of Fe^2+^ into root cells by Fe^2+^ transporters, including ZIPs [12]. Furthermore, phenolics are secreted to facilitate iron acquisition during Fe starvation. These substances serve as iron chelators or electron donors for Fe^3+^ reduction during iron uptake [16]. Graminaceous plants such as rice and corn use strategy II, which involves the synthesis and secretion of phytosiderophores (PS) to facilitate iron uptake. The chelated Fe^3+^ is then translocated into roots cell by YS proteins. The transportation of iron in graminaceous plants is mediated by NA and YSL proteins. These results indicated that ZIPs play essential roles in both absorption and translocation of Zn and Fe in nongraminaceous plants, while their functions in graminaceous plants remain less understood.

ZIPs function in ion homeostasis as they can transport cations into the cytoplasm [17]. *AtIRT1* was the first cloned member of ZIP family and encodes a major Fe transporter at the root surface [18–21]. Further studies showed that *irt1* had lower Ni accumulation than the wild-type plants under Fe-deficiency status, indicating that AtIRT1 play essential roles in Fe and Ni translocation in *Arabidopsis* [22]. AtIRT2 localized to intracellular vesicles, suggesting it may play roles in compartmentalisation and remobilising of iron into internal storage vesicles to avoid metal toxicity [23]. Additionally, the accumulation of Zn in reproductive organs correlate with expression levels of *VvZIP3* during the reproductive growth stage, indicating that ZIPs may be associated with Zn distribution in embryo and endosperm [6]. It has also been reported that overexpression of *ZIPs* may result in elevated metal ion content. Overexpressing *AtZIP1* in *Hordeum vulgare* increases the Zn and Fe contents in seeds [24]. Similarly, Fe and Zn levels were increased in shoots, roots and seeds when overexpressing *OsIRT1* in rice [25]. However, overaccumulating *OsZIP4*, result in increased Zn contents in roots, while Zn levels in seeds was significant lower than in non-transgenic plants [26]. Since it was reported that *OsZIP4* was expressed in the phloem cells of stem, and vascular bundles of leaves and roots [27], it can be assumed that the endogenous expression profiles of *ZIP* genes may be critical for appropriate Zn and Fe distribution and metal homeostasis in plants.

The contribution of ZIP genes in the uptake and translocation of iron and zinc has been investigated extensively in *Arabidopsis* and rice. However, our understanding of the functions of ZIPs in maize remains limited. In this study, two ion transporters, *ZmIRT1* and *ZmZIP3*, were cloned from maize and showed distinctive expression patterns. It was demonstrated that ZmIRT1 and ZmZIP3 showed different activity of yeast complementation, and the expression level of *ZmIRT1* was induced by Fe deficiency [28]. To explore the physiological function of ZmIRT1 and ZmZIP3 in plants, transgenic *Arabidopsis* lines overexpressing *ZmIRT1* and *ZmZIP3* were generated. The contents of Zn and Fe in transgenic plants were then measured using ICP-OES and Zinpyr-1 staining. We analysed the phenotype of overexpression lines under excess and deficient Zn and Fe conditions, and detected the transcription levels of key genes that play a role in the uptake and translocation of Zn and Fe in transgenic *Arabidopsis* plants. It was shown that ZmIRT1 and ZmZIP3 are functional metal transporters, and ectopic overexpression of *ZmIRT1* stimulated endogenous Fe uptake mechanisms, which may facilitate metal uptake and homeostasis.

## Materials and Methods

### Plant materials and growth conditions

The plants of maize (*Zea mays* L.) inbred line B73 were grown in the greenhouse. For expression analysis of *ZmIRT1* and *ZmZIP3*, root, stem, leaf, and sheath samples were collected from the flare opening stage; tassel, anther, ear, cob, silk, and husk leaf were collected during the flowing period, and embryos and endosperms were sampled at 10, 15, and 20 d after pollination.

Seeds of *Arabidopsis* (Columbia wild-type and transgenic lines) were surface-sterilised with 75% ethanol for 10 min and grown on the standard Murashige and Skoog (MS) medium containing 30 μM ZnSO_4_, 100 μM Fe^3+^-EDTA, 0.1 μM CuSO_4_, and 10 μM MnSO_4_ as micronutrients [29]. For metal nutrition deficiency and excess analysis, seeds were germinated and grown for 13 d on standard MS and MS medium lacking ZnSO_4_ (Zn-deficient) or Fe^3+^-EDTA (Fe-deficient), and with 200 μM ZnSO_4_ (Zn-excess) or 300 μM Fe(III)-EDTA (Fe-excess). Surface-sterilised seeds were germinated on agar plates, and vernalisation was performed at 4°C for 2d. Seedlings were grown on MS medium for 10 d at 22°C under a light: dark cycle of 16 h: 8 h, after which the seedlings were transplanted to soil in a glasshouse.

To analyse the zinc and iron contents of wild-type and transgenic plants, the shoots and roots were sampled, respectively, at the bolting stage. To determine the zinc and iron levels in seeds, the same batch of seedlings was used.

### Subcellular localization

For subcellular localization, the GFP-fusion expression vector was constructed as described in detail previously [28]. The ZmIRT1 and ZmZIP3-GFP fusion plasmid and the mcherry labelled ER marker were co-transformed into maize mesophyll protoplasts, respectively, following procedures described previously [30, 31]. After 14 h of incubation in the dark at 26°C, the GFP and ER fluorescence was determined by a confocal microscope (LSM700; Carl Zeiss).

### Plasmid construction, *Arabidopsis* transformation, and overexpression plant confirmation

To construct *ZmIRT1* and *ZmZIP3*-overexpressing vectors, the *ZmIRT1* and *ZmZIP3* coding sequences were amplified by PCR using the gene-specific primers IRT1oxF (with an added *Xba*I site) and IRT1oxR (with an added *SnaB*I site) for *ZmIRT1*, and ZIP3oxF (with an added *Sma*I site) and ZIP3oxR (with an added *Kpn*I site) for *ZmZIP3* (S1 Table), respectively. The PCR fragments were cloned into the corresponding site of the pBI121 vector to generate pBI121-*ZmIRT1* and pBI121-*ZmZIP3*. *Agrobacterium tumefaciens* strains GV3101 harbouring the overexpression plasmids were used to transform *Arabidopsis* and generate the transgenic lines. Transformation was performed following the floral dip protocol [32]. Overexpression plants were confirmed by both PCR analysis and kanamycin selection.

### Metal element analysis

For elemental analysis, leaves and roots were harvested, and the processing method as described in detail previously [33]. The tissues and seeds were air-dried for 6 d at room temperature. For elemental measurement, 0.5 g of plant material was digested in 2-mL HNO_3_ overnight, after which 2 mL of H_2_O_2_ were added, followed by microwave digestion. When digestion was completed, and the digests were diluted with Millipore water and filtered. The volume was then adjusted to 25 mL and analysed by ICP-OES on an iCAP 6000 Series spectrometer (Thermo-Fisher). For metal element analysis, three biological replicates were used, for each of which three technical replicates were performed. This experiment was performed at the Analysis and Testing Center of Tsinghua University.

### Zinpyr-1 staining and quantification of fluorescence

The zinc concentration in various tissues of *Arabidopsis* was examined using Zinpyr-1, which is a cell-permeable and fluorescent probe that selectively detects free zinc in living cells, and propidium iodide was used to stain cell walls. The Zinpyr-1 and propidium iodide working solution were diluted to 20 μM and 75 μM, respectively. The 11 d old seedlings were collected and washed in deionised water, then immersed in Zinpyr-1 and propidium iodide to stain. Preparation and staining process of Zinpyr-1 and propidium iodide were performed as described in detail previously [33]. Images were obtained using a confocal microscope (LSM700; Carl Zeiss) with 488-nm excitation. To compare the signal intensity of transgenic and wild-type lines, all imaging parameters were fixed.

A method of signal quantification was developed using ZEN 2009 Light Edition software. For comparative analysis of zinc content in shoots, leaves and roots, the green and red fluorescence intensities in corresponding areas of the wild-type and transgenic plants were selected and calculated. The ratio of green-to-red fluorescence was used to normalize the results.

### Reverse transcription (RT)-PCR and quantitative RT-PCR analysis

Total RNA was extracted using TRIzol (TransGen) from wild-type and transgenic lines, as well as various organs from the maize inbred line B73. For cDNA synthesis, 4 μg total RNA were reverse transcribed to cDNA in a 40-μL reaction volume using One-step gDNA Removal and cDNA Synthesis SuperMix (Transgen) and incubated at 42°C for 30 min in a H2O3-PRO drybath (Coyote Bioscience). The gene-specific primers (S1 Table) were used for RT-PCR and quantitative RT-PCR. The PCR mix and PCR conditions were performed as described in detail previously [28]. Data were analysed using the ABI7500 software (version 2.0.5) via the 2^-ΔΔCT^ method, and the expression levels of *ZmActin1* and *AtUBP6* were used as references in maize and *Arabidopsis*, respectively. For quantitative RT-PCR analysis, three biological replicates were used, for each of which three technical replicates were performed.

## Results

### *ZmIRT1* and *ZmZIP3* show different gene expression patterns but similar subcellular localizations

**Fig 1:**
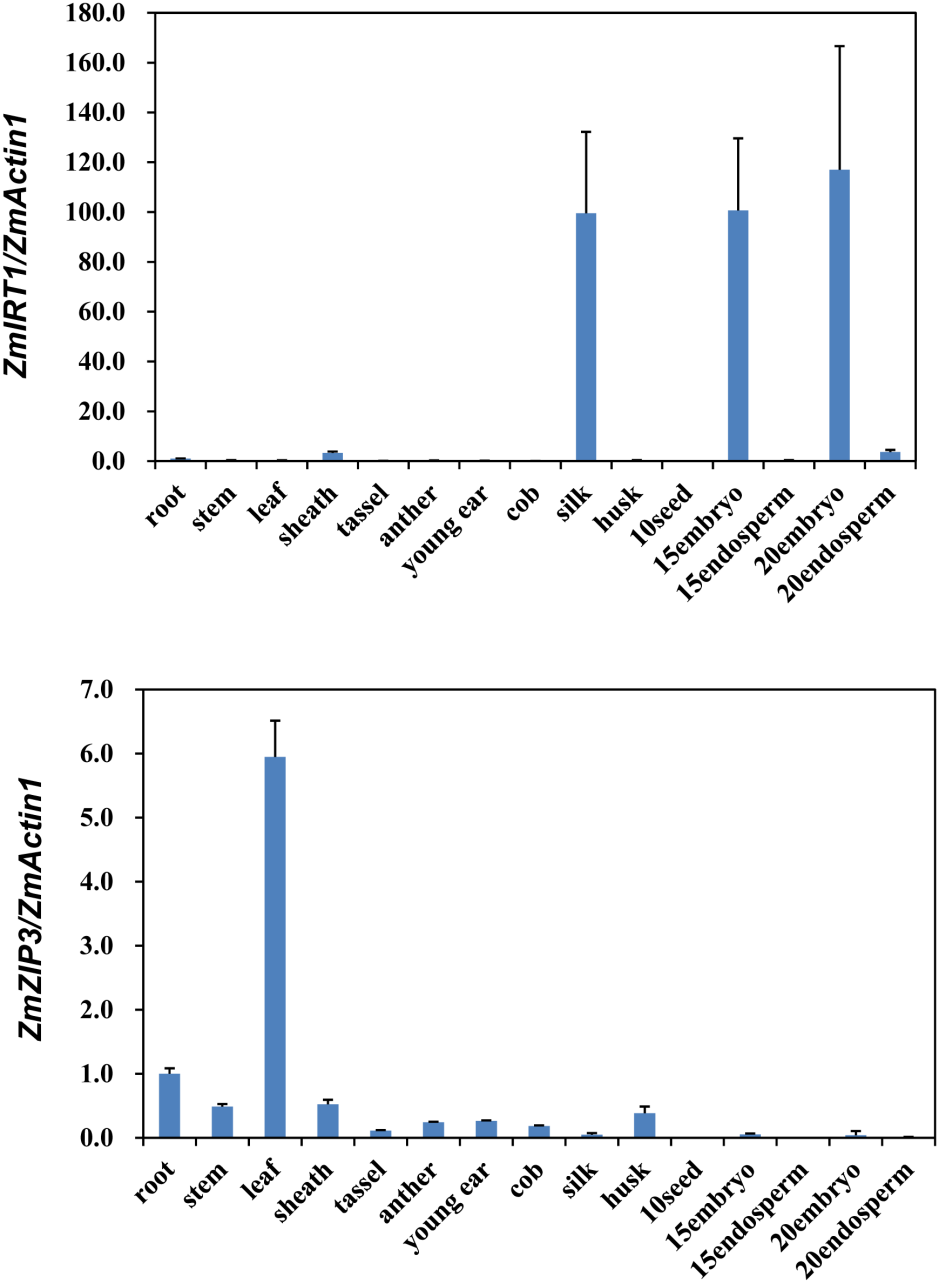
Expression levels of *ZmIRT1* and *ZmZIP3* in maize organs. The relative expression levels of *ZmIRT1* and *ZmZIP3* were normalised to that of *ZmActin1*. quantitative RT-PCR data were analysed following the 2^-ΔΔCt^ method. Error bars indicate standard deviations.

In the present study, two *ZmZIP* genes were identified and showed distinctive expression patterns. Quantitative RT-PCR was applied to determine the transcript levels of *ZmIRT1* and *ZmZIP3* in various organs and developing seeds. *ZmIRT1* was predominantly expressed in silk and embryo, while *ZmZIP3* was expressed in a leaf-specific manner (Fig 1). The subcellular localization of ZmIRT1 and ZmZIP3 may be useful for inferring their functions in maize. Thus, the fusion construct was co-transferred with an ER marker and expressed transiently in maize protoplasts (S1 Fig). We found that ZmIRT1-GFP and ZmZIP3-GFP fusion proteins were localized to the plasma membrane and endoplasmic reticulum, as observed previously in *Arabidopsis* protoplasts.

### Transgenic *Arabidopsis* ectopically expressing *ZmIRT1* and *ZmZIP3* respond differently to metal excess and deficiency conditions

**Fig 2:**
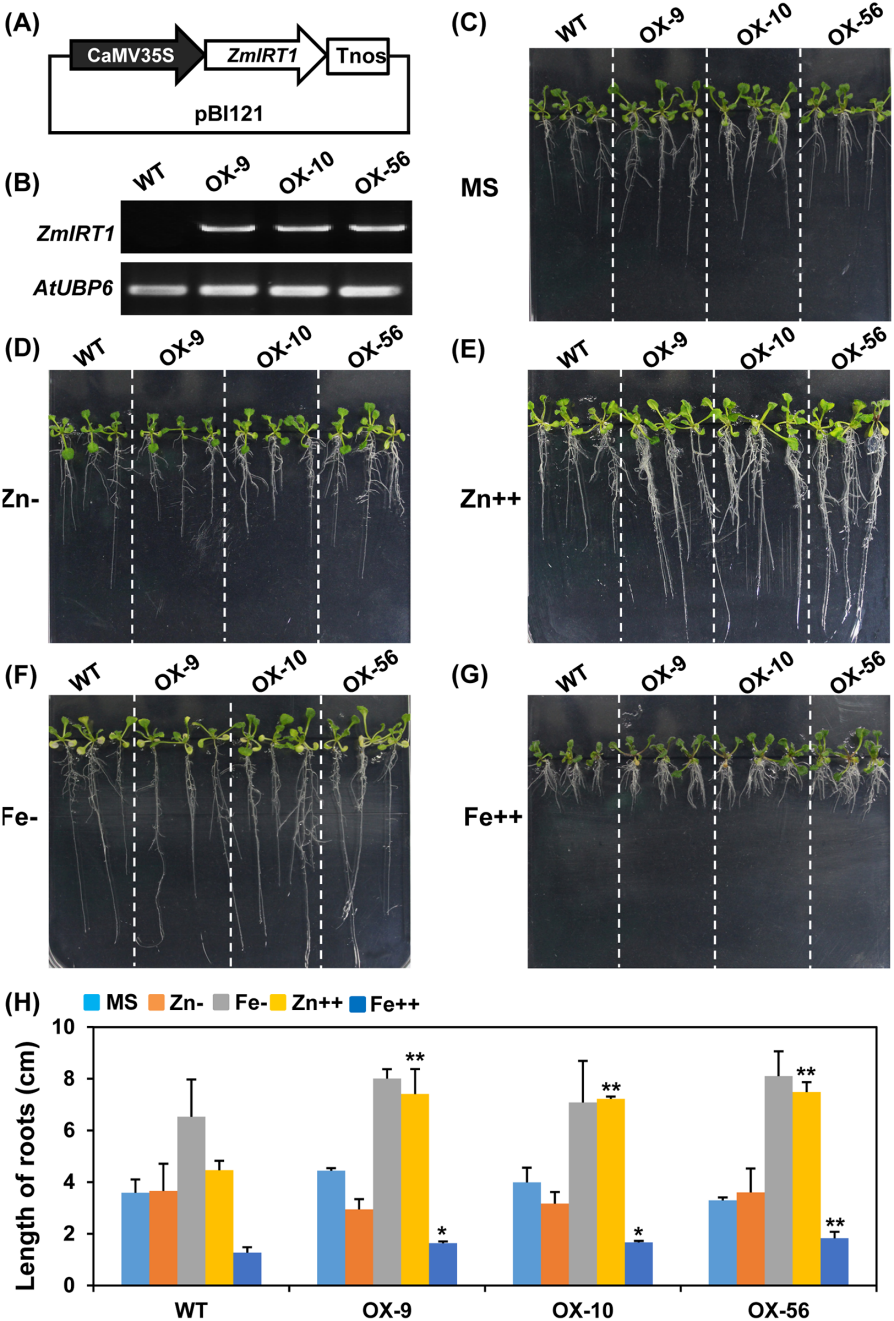
Phenotypic analysis of the *ZmIRT1*-overexpressing *Arabidopsis* (OX9, OX10, OX56) and wild-type (WT) plants under various metal conditions. (A) Schematic diagram of pBI121-*ZmIRT1* construct. (B) RT-PCR verifying the expression of *ZmIRT1* in transgenic lines. *AtUBP6* was used as an internal control. The growth trend of 2-week-old seedlings under (C) standard nutrient conditions (MS medium), (D) Zn, and (F) Fe-deficiency (Zn- and Fe-), as well as (E) 200 μM ZnSO_4_ (Zn++) and (G) 300 μM FeSO_4_ (Fe++) treatment. (H) The root lengths of the wild-type and transgenic plants were measured using the ImageJ software. Error bars represent standard errors. Asterisks denote significant differences: *P<0.05, **P<0.01.

**Fig 3:**
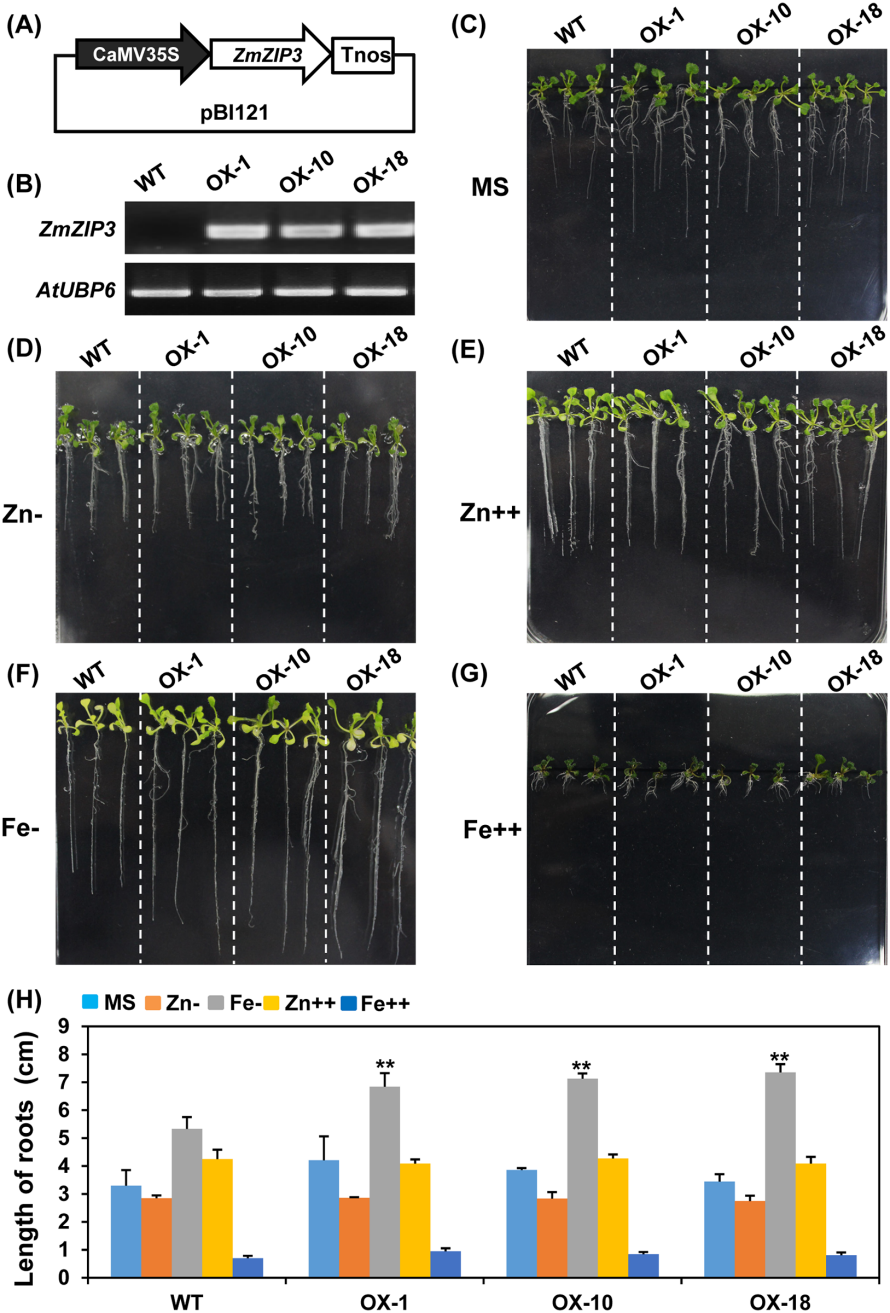
Phenotypic analysis of the *ZmZIP3*-overexpressing *Arabidopsis* (OX1, OX10, and OX18) and wild-type (WT) plants under various metal conditions. (A) Schematic diagram of the pBI121-*ZmZIP3* construct. (B) RT-PCR analysis of *ZmZIP3* overexpression lines. *AtUBP6* was amplified to show equal amounts of RNA in each sample. The growth trend of 2-week old seedlings under (C) standard nutrient condition (MS medium), (D) Zn, and (F) Fe-deficiency (Zn- and Fe-), as well as (E) 200 μM ZnSO_4_ (Zn++) and (G) 300 μM FeSO_4_ (Fe++) treatment. (H) The root lengths of the wild-type and transgenic plants were measured using the ImageJ software. Error bars represent standard errors. Asterisks denote significant differences: *P<0.05, **P<0.01.

It has been demonstrated that ZmIRT1 has a high selectivity for iron transportation, while ZmZIP3 is a zinc transporter. Additionally, *ZmIRT1* and *ZmZIP3* exhibited distinct expression patterns in response to excess and deficient metal conditions [28]. Since *ZmIRT1* and *ZmZIP3* have different spatial and temporal expression profiles, they were selected for further functional characterisation. The transgenic *Arabidopsis* ectopically overexpressing *ZmIRT1* and *ZmZIP3* were generated, respectively. To verify the expression of *ZmIRT1* and *ZmZIP3*, their transcript levels were determined using RT-PCR in transgenic and wild-type plants (Figs 2A, 2B, 3A and 3B). *ZmIRT1* lines OX9, OX10, and OX56 and *ZmZIP3* lines OX1, OX10, and OX18 were chosen for further research. To examine the effect of *ZmIRT1* or *ZmZIP3* on plant growth and mineral nutrition status, we tested the phenotype of transgenic and wild-type *Arabidopsis* on medium with various metal compositions. No significant growth difference was observed between *ZmIRT1*-overexpressing and wild-type plants under sufficient and deficient metal nutritional conditions (Fig 2C, 2D, 2F and 2H). However, in response to Fe and Zn-excess, the roots of *ZmIRT1* transgenic plants were longer than those of the wild-type (Fig 2E, 2G and 2H), suggesting that the overexpressing plants may tolerate excess metals. On the contrary, *ZmZIP3*-overexpressing transgenic plants were resistant to iron deficiency since their roots were longer than those of wild-type plants (Fig 3).

### Overexpression of *ZmIRT1* and *ZmZIP3* affects Fe and Zn distribution in plants

**Fig 4:**
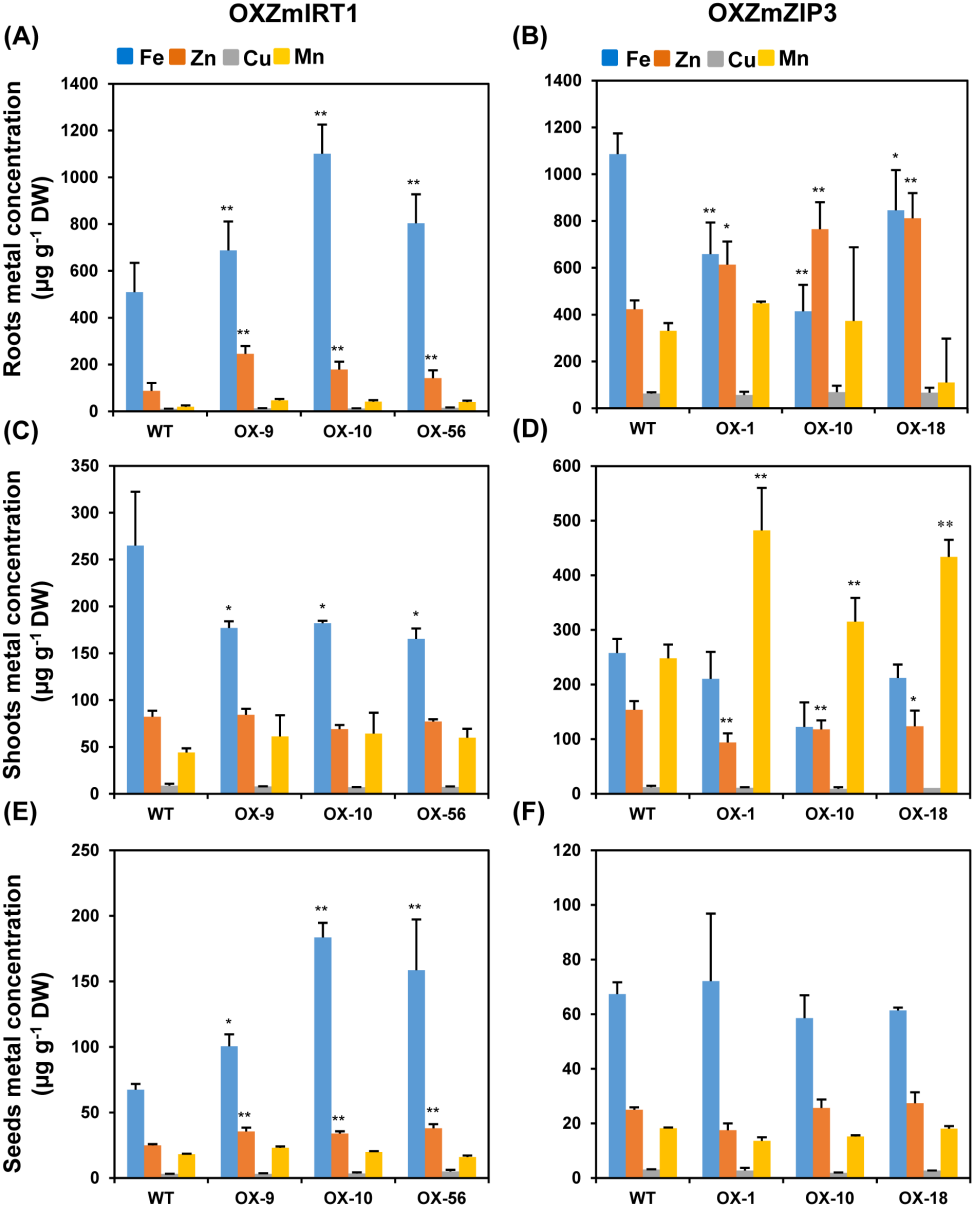
Metal contents in wild-type (WT) and overexpression lines (OXZmIRT1 and OXZmZIP3). Plants were cultivated in nutrient soil. The Fe, Zn, Cu, and Mn contents of roots (A, B), shoots (C, D) and mature seeds (E, F) were analysed using ICP-OES. Values represent the means ± SD (n = 15). Asterisks indicate significant differences: *P<0.05, **P<0.01.

To assess whether overexpression of *ZmIRT1* or *ZmZIP3* affects metal distribution or accumulation, we measured the contents of zinc and iron in transgenic plants at the bolting stage and in mature seeds. When grown on a metal-sufficient medium, the *ZmIRT1*-overexpressing transgenic plants accumulated more iron (34.87–115.95%) and zinc (61.97–179.5%) in roots, as well as enhanced iron (49.29–172.57%) and zinc (35.69–51.7%) in seeds compared with wild-type plants (Fig 4A and 4E). However, the accumulation of iron in shoots was decreased and zinc content was almost unchanged in transgenic plants expressing *ZmIRT1* (Fig 4C). In addition, Cu and Mn concentrations did not differ significantly between *ZmIRT1*-transgenic and wild-type plants (Fig 4A, 4C and 4E). These results indicated that *ZmIRT1* facilitated Zn and Fe uptake in roots and may be involved in the translocation of Zn and Fe into seeds.

Compared with wild-type plants, *ZmZIP3*-overexpressing transgenic plants contained more Zn (45.13–92.14%) in roots and Mn (27.00–94.3%) in shoots, although the levels of Fe in roots and Zn in shoots were decreased (Fig 4B and 4D). Moreover, no obvious changes were detected in metal concentrations in the seeds of *ZmZIP3*-overexpressing lines (Fig 4F). These results indicated that *ZmZIP3* may be associated with Zn uptake from soil to root, and ectopic over-accumulation of *ZmZIP3* in the root may disrupt the local distribution of metal transporters and result in decreased Fe and Zn concentrations in roots and shoots, respectively.

### Histological zinc accumulation patterns were examined in *ZmIRT1*- and *ZmZIP3*-overexpressing plants

**Fig 5:**
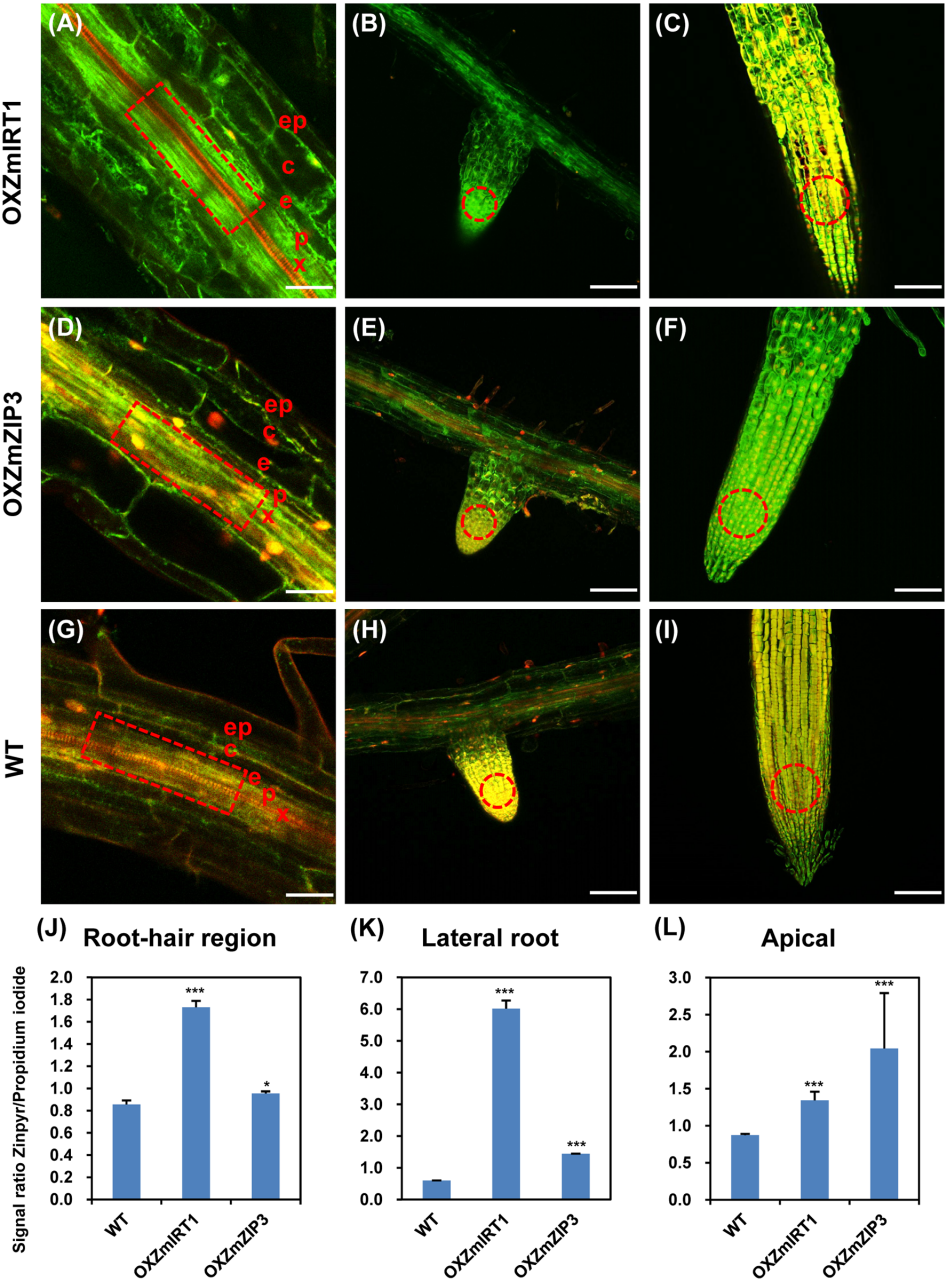
Confocal laser-scanning microscopy (CLSM) images of root-hair region, lateral root, and apical root of the overexpression and wild-type (WT) plants stained with Zinpyr-1. Roots of wild-type and overexpression plants were successively stained with Zinpyr-1 and propidium iodide to visualise Zn (green) and cell walls (red), respectively. (A-C) and (D-F) show *ZmIRT1* (OXZmIRT1) and *ZmZIP3* (OXZmZIP3) overexpression plants, respectively, and (G-I) show the wild-type. The boxed regions in (A, D, G) and circled regions in (B, C, E, F, H, I) show the regions used for quantifying average fluorescence intensity. Bars represent 100 μm. (J-L) show the fluorescence intensity of selected regions in the root-hair region, lateral root, and apical root, respectively. The signal ratio of Zinpyr-1/ propidium iodide represents the relative content of Zn. Values represent means ± SD (n = 6). x, xylem. p, pericycle. e, endodermis. c, cortex. ep, epidermis. Asterisks indicate significant differences from the wild-type mean: ***P < 0.001, *P < 0.05.

**Fig 6:**
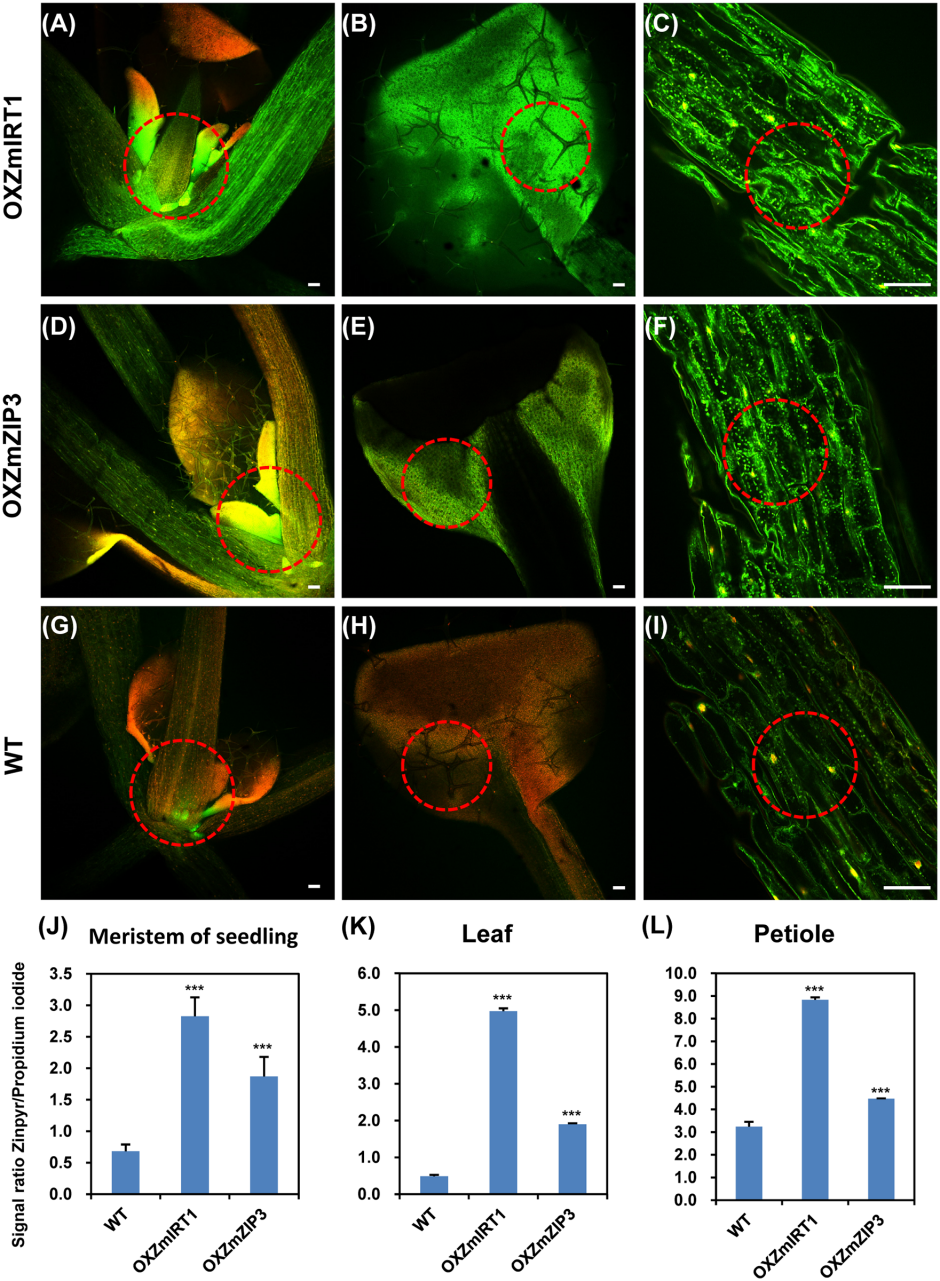
Confocal laser-scanning microscope (CLSM) images of seedlings, leaves and petioles of the overexpression and wild-type (WT) plants stained with Zinpyr-1. Shoots of wild-type and overexpression plants were consecutively stained with Zinpyr-1 and propidium iodide to visualise Zn (green) and cell walls (red), respectively. (A-C) and (D-F) show *ZmIRT1* (OXZmIRT1) and *ZmZIP3* (OXZmZIP3) overexpression plants, respectively, and (G-I) show the wild-type. The circled regions in (A-I) illustrate the regions used to quantify average fluorescence intensity. Bars represent 100 μm. (J-L) show the fluorescence intensity of selected regions of seedlings, leaves, and petioles, respectively. The signal ratio of Zinpyr-1/ propidium iodide represents the relative content of Zn. Values represent the means ± SD (n = 6). Asterisks indicate significant differences from the wild-type mean: ***P < 0.001.

To visualise the histological localization of Zn in roots and shoots, 11 d old seedlings of wild-type and transgenic plants were immersed in 20 μM Zinpyr-1 and 75 μM propidium iodide, and the signal was examined using a confocal laser-scanning microscope. The fluorescence intensity was calculated using the ZEN software, and the green/red ratio was applied to indicate the Zn relative quantities. In roots of transgenic plants, a high fluorescence signal level was observed in the xylem, while that in pericycle cells was relatively weak (Fig 5A, 5D and 5G). This result is consistent with previous reports [34]. In addition, the fluorescence signals in roots of wild-type plants were considerably weaker than that in transgenic lines (Fig 5J–5L). Moreover, the Zinpyr-1-dependent fluorescence signal in the meristem zone of lateral root and the root apical was significantly higher than that in the elongation region in both wild-type and overexpression lines (Fig 5), suggesting that Zn may be essential for the emergence and development of lateral roots. We also observed that fluorescence signals in the meristem of seedling, leaves, and petioles of overexpression plants were significantly higher than those in non-transgenics (Fig 6J and 6L). These results showed that transgenic plants accumulated more Zn than did the wild-type during seedling development. Moreover, Zn is concentrated in the xylem of roots and petiole of leaves where Zn flow is activated, as well as in the meristem of seedlings and roots where high levels of Zn may be required for bud development.

### Expression levels of key genes involved in metal uptake and transportation were altered in transgenic plants

**Fig 7:**
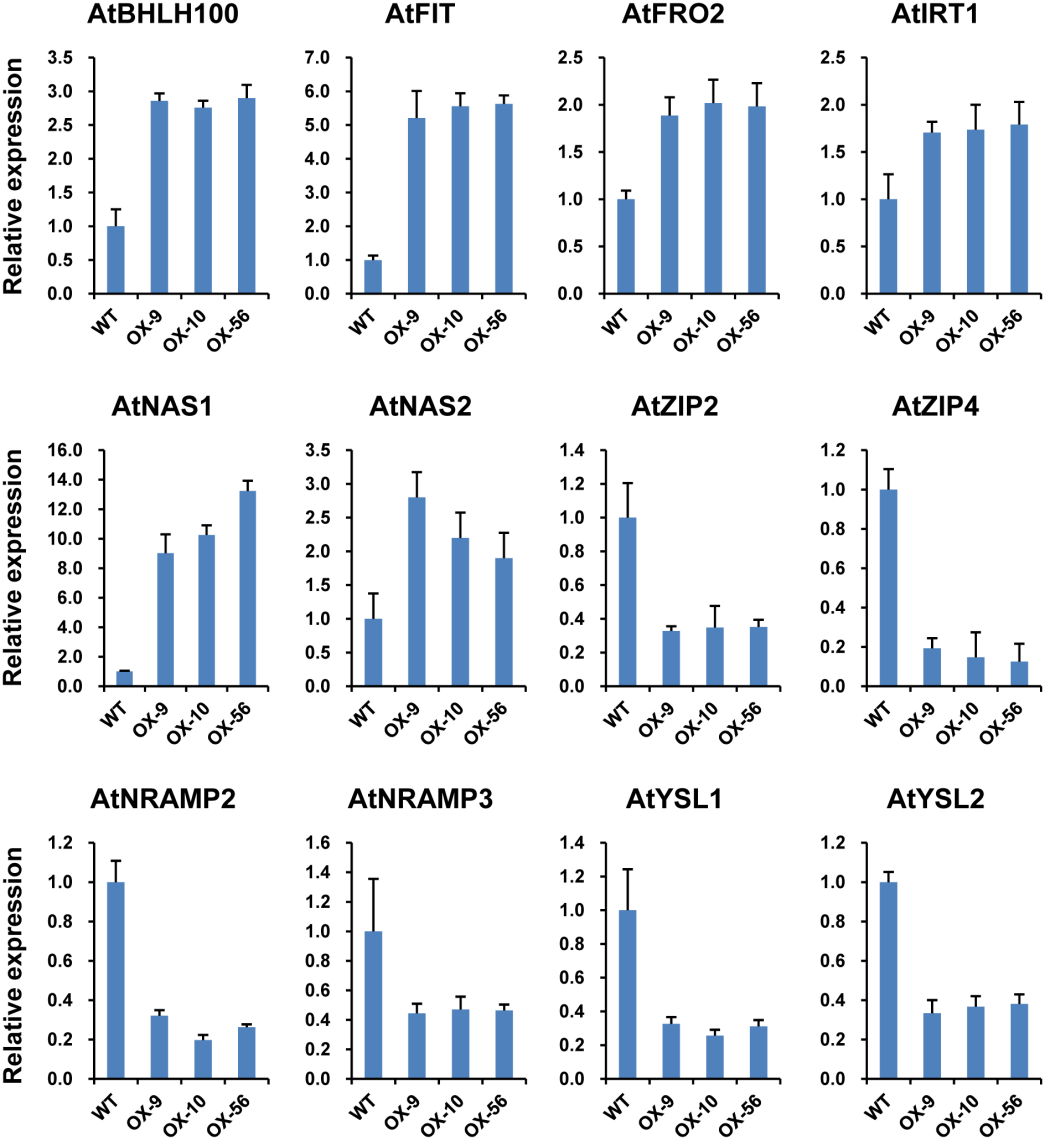
Expression of genes involved in metal uptake and translocation in wild-type (WT) and *ZmIRT1*-overexpressing lines (OX9, OX10, and OX56). RNA of wild-type and transgenic plants was prepared from 13 d old seedlings grown on standard MS medium. Relative mRNA abundance of each gene was normalised to that of *AtUBP6*. Data from quantitative RT-PCR were analyzed following the 2^-ΔΔCt^ method. Error bars indicate standard deviations.

**Fig 8:**
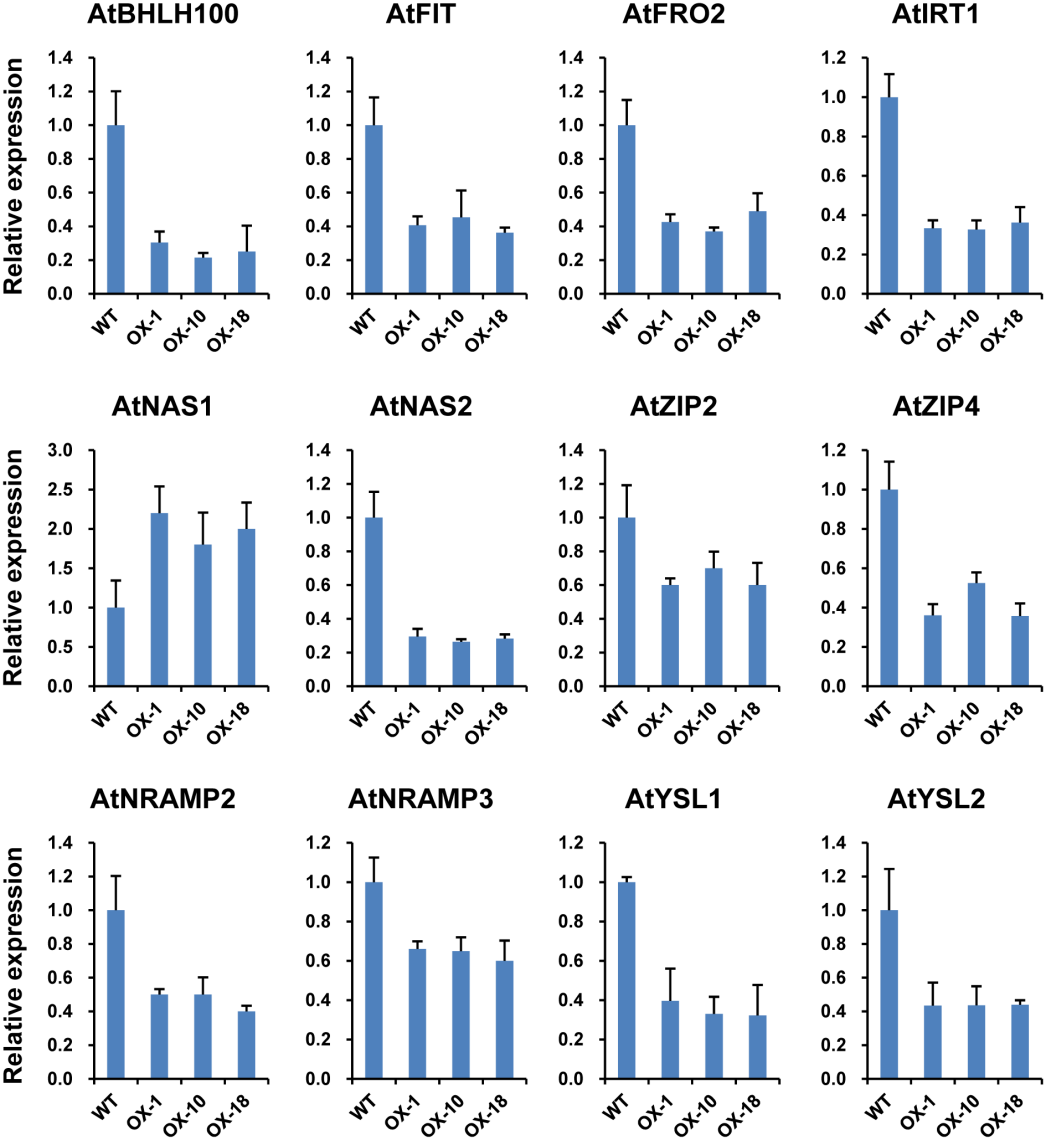
Expression of genes involved in metal uptake and translocation in wild-type (WT) and *ZmZIP3*-overexpressing lines (OX1, OX10, and OX18). RNA of wild-type and transgenic plants was extracted from 13 d old seedlings grown on standard MS medium. Relative mRNA abundance of each gene was normalised to that of *AtUBP6*. Data from quantitative RT-PCR were analyzed following the 2^-ΔΔCt^ method. Error bars indicate standard deviations.

Since the Zn accumulation patterns were affected and sensitivities to various Zn/Fe conditions were altered in the *ZmIRT1* and *ZmZIP3* overexpression lines, it can be assumed that the Zn and Fe homeostasis in the transgenic lines had changed. For genes involved in metal uptake and transportation, *FRO2* (*Ferric Reductase Oxidase*) and *IRT1* (*Iron-Regulated Transporter*), as well as several members of the *NAS*, *YSL*, and *ZIP* family were investigated. The results showed that the expression levels of these genes were changed in the transgenic lines. In *ZmIRT1* overexpressing plants, the expression of *FRO2*, *IRT1*, and *NAS* family genes were enhanced (Fig 7), while the endogenous genes involved in metal transportation were repressed, including *YSL1* and *YSL2* among *YSLs* and *ZIP2* and *ZIP4* from the *ZIP* family (Fig 7). Unlike in the *ZmIRT1* transgenic lines, the expression of the above genes was down regulated, excluding *NAS1* in *ZmZIP3*-overexpressing plants (Fig 8).

For expression analysis of transcription regulators involved in metal uptake and other intracellular transporters, *bHLH* (*basic-helix-loop-helix*) and *FIT* (*FER-like iron-deficiency-induced transcription factor*), which control the expression of *FRO2* and *IRT1* [35–38], as well as *NRAMP3* (natural resistance-associated macrophage protein) and *NRAMP4* responsible for transporting Fe^2+^ from the vacuole to cytoplasm [39, 40] were examined. It was found that *BHLH100* and *FIT* were induced (Fig 7), whereas the expression of *NRAMP 2* and *3* was repressed (Fig 7). These results indicated that the Zn and Fe uptake system is activated in *ZmIRT1* transgenic plants, while intracellular metal transportation is suppressed. In the *ZmZIP3*-overexpressing lines, expression of the above-mentioned genes was downregulated (Fig 8), suggesting that Zn/Fe uptake and intracellular transportation are inactivated.

## Discussion

In this study, we demonstrated that ZmIRT1 and ZmZIP3 are involved in the uptake and translocation of metal ions in plants, and our results suggested that they might be associated with metal homeostasis by regulating the expression of genes involved in metal uptake and transportation.

It was reported that transgenic plants overexpressing *OsIRT1* exhibited less chlorosis and had higher chlorophyll content than the wild-type under Fe-deficient conditions. Moreover, the Fe content was also increased in mature seeds of *OsIRT1* overexpression plants [25]. In addition, overexpression of *MxIRT1* enhanced Fe and Zn contents in rice seeds [41]. Likewise, overexpression of *AtZIP1* in barley lead to an elevated level in the short-term Zn uptake, and enhanced Zn and Fe contents in seeds [24]. However, overexpression of *OsZIP5* and *OsZIP8* in rice leads to increased Zn levels in roots, although the Zn content was reduced in shoots and mature seeds of overexpression lines [29, 42]. These data suggested that the metal transportation activity of ZIP proteins may differ, or ectopic accumulation of ZIPs may disrupt the endogenous metal homeostasis due to incongruous *in vivo* ion transporter gradients. It was found that *ZmIRT1* and *ZmZIP3* showed strongly and relatively weaker reverse the yeast complement activity. Moreover, the expression of *ZmIRT1* was significantly upregulated in roots and shoots under Fe-deficiency, and it was induced in shoots at 96 h after Zn-deficiency. These results suggested that ZmIRT1 might be essential for both uptake and translocation of Fe and Zn. On the contrary, *ZmZIP3* was induced by Zn deficiency, indicating that ZmZIP3 may be important for Zn uptake [28]. In this study, we examined the expression pattern of *ZmIRT1* and *ZmZIP3* in various organs. *ZmIRT1* was predominantly expressed in silk and embryo, while *ZmZIP3* is a leaf-specific gene. The different expression pattern of *ZmIRT1* and *ZmZIP3* indicated that they might play distinct roles in metal uptake and translocation. To analyse the physiological roles of the *ZmIRT1* and *ZmZIP3* in plants, we generated transgenic *Arabidopsis* and examined whether ZmIRT1 and ZmZIP3 could transport Zn or Fe in plants. It was found that overexpression of *ZmIRT1* in *Arabidopsis* enhanced Fe and Zn contents in both roots and seeds (Fig 4A and 4E), suggesting that ZmIRT1 is a functional Fe and Zn transporter, and it may be associated with the translocation of metals toward seeds. Additionally, we found that *ZmIRT1*-overexpressing plants grew longer roots than the wild-type under Zn- and Fe-excess conditions (Fig 2E and 2G), which indicates that the transgenic plants are more tolerant to excess zinc and iron. ZmIRT1 is localized to the plasma membrane and endoplasmic reticulum in maize (S1 Fig) and *Arabidopsis* mesophyll protoplasts [28]. Therefore, ZmIRT1 may function in translocating excess subcellular free Fe^2+^ and Zn^2+^ into the endoplasmic reticulum. Moreover, ZmIRT1 may play an essential role in storage and detoxification of Zn and Fe. On the contrary, *ZmZIP3*-overexpression plants developed more lateral roots and longer roots than wild-type under Fe-deficient conditions (Fig 3F), indicative of a stronger Fe deficiency response in transgenic plants. Consistently, the Fe content in roots was decreased in *ZmZIP3*-overexpressing plants, although Zn accumulated in roots (Fig 4B). It is noteworthy that the growth of *ZmZIP3* transgenic plants was better than the wild-type, despite the Fe-deficiency response in roots (Fig 3F). This is probably because over-accumulation of *ZmZIP3* facilitated the uptake of Zn in the roots, which alleviated the symptoms of Fe deficiency.

Besides the morphological changes, the expression of many genes associated with Fe/Zn deficiency and excess responses were also altered in the *ZmIRT1*- and *ZmZIP3*-overexpressing plants. It was reported that the expression profiles of genes associated with Fe and Zn uptake and translocation were regulated in accordance with the internal and environmental metal conditions. *OsNAS3*, *OsNAAT1*, and *OsDMAS1* encode enzymes for the biosynthesis of NA and 2’-deoxymugineic acid (DMA), which plays important roles in chelating and distributing metal ions within plants. The expression of *OsNAS3*, *OsNAAT1*, and *OsDMAS1* are enhanced in the overexpression lines under Zn-deficient conditions [29]. In addition, the activation of *OsNAS3* resulted in increased Zn and Fe contents in rice grains, suggesting that *OsNAS3* contributes to ion homeostasis [43]. In our study, the transcription levels of genes associated with Zn/Fe uptake and transportation may reflect the endogenous metal status; thus, we investigated the expression of 12 *Arabidopsis* genes known to be involved in the homeostasis of Zn and Fe (Fig 7). The results showed that the expressions of genes involved in the uptake of Fe and Zn were altered in both *ZmIRT1* and *ZmZIP3* transgenic lines. The transcript levels of *AtNAS1*, *2*, which are associated with NA synthesis, *AtFRO2* that plays a role in ferric reduction, and *AtIRT1* that is related to ferrous uptake were up-regulated in *ZmIRT1*-overexpressing plants (Fig 7). The enhanced expression of these genes may be due to the root absorbed too much iron that stimulated the expression of *NAS*, *FRO* and *IRT1*, which associated with iron uptake. In contrast, *AtFRO2* and *AtIRT1* were reduced in the *ZmZIP3* transgenic lines (Fig 8), possibly due to the iron content was decreased so suppressed the expression of these genes. These results suggest that ZmIRT1 and ZmZIP3 play different roles in metal homeostasis. The stimulation of these genes in *ZmIRT1*-overexpressing lines may be due to the increased expression of transcription factors that regulate ion homeostasis. It has been demonstrated that Fe homeostasis is controlled by conserved transcriptional networks in both grasses and non-grasses [44, 45]. Under Fe-deficiency, FIT (FER-like iron-deficiency-induced transcription factor) interacts with bHLH038 and bHLH039 to regulate the expression of *IRT1* and *FRO2* in *Arabidopsis* [37]. It was also reported that *FIT*, *IRT1*, *FRO2*, and *BHLH100* are iron deficiency induced genes in roots [15]. Although there are no orthologs of *FIT* in rice, *OsIRO2* is a regulator of the Fe deficiency-responsive gene, which is highly similar to bHLH38/39. OsIRO2 regulates the expression of Fe^3+^-PS translocation-related genes, with the exception of OsIRT1 [46]. Co-overexpression of *FIT* and *bHLH38* or *bHLH39* results in increased expression of *NAS1* and *NAS2*, which enhanced NA accumulation and increased transport of Fe from roots to shoots [47]. We found that the expression of *FIT* was stimulated in the *ZmIRT1* transgenic plants, indicating that a transcriptional cascade was activated as a result of ectopic accumulation of ZmIRT1. Besides, pleiotropic effects may also contribute to the activation of gene expression since a greater quantity of Fe and Zn was absorbed into the cytoplasm when *ZmIRT1* was overexpressed. The expression of *NRAMPs*, *YSLs*, and endogenous *ZIPs* were suppressed in both *ZmIRT1* and *ZmZIP3* lines (Figs 7 and 8), suggesting that over-accumulation of ZIP proteins may inhibit the expression of endogenous genes that function in metal transportation. Taking this into consideration, our results suggested that overexpressing *ZmIRT1* may facilitate Zn and Fe uptake by inducing genes associated with strategy I (ferric reduction and ferrous uptake) and strategy II (ion-chelator synthesis) iron uptake systems, although the expression of genes involved in intercellular ion transportation was suppressed.

Fe uptake mechanisms were classified into two strategies. Strategy I, a reduction-based strategy, is used by non-grasses such as *Arabidopsis* [44, 45]. It begins with acidification of the rhizosphere and increases the solubility of ferric in the soil [48–50]. The reduction of ferric irons then occurs on the membrane by a membrane-bound ferric-chelate reductase AtFRO2, and ferrous is taken into root epidermal cells by transmembrane transporters, such as AtIRT1 [21, 22]. On the contrary, strategy II, a chelation-based strategy is used by grasses such as barley, rice, and maize. Phytosiderophores are released into the rhizosphere to chelate ferric [51], and Fe^3+^-PS can be absorbed into root cells by YSLs [52, 53]. The main distinction between these two strategies is different oxidation and reduction forms of iron absorbed into the root cells, as non-grasses prefer ferrous and grasses absorb ferric. It is generally considered that the iron uptake mechanisms used by these two strategies do not overlap. The identification of two functional Fe^2+^ transporters, OsIRT1 and OsIRT2, indicates that strategy I may be used by graminaceous plants [54, 55]. It was proposed that rice uses a combined strategy including strategy II and partial strategy I to uptake Fe from the soil. TOM1/OsZIFL4 and OsYSL15 were reported to be involved in the release of PS and uptake of Fe^3+^-PS as strategy II, while OsIRT1 translocated Fe^2+^ into root cells through strategy I. However, the genes responsible for acidification and ferric reduction seemed to be lost in the rice genome [54, 56, 57]. As a graminaceous plant, maize was considered to apply strategy II for Fe uptake. However, ZmIRT1 was identified as a functional Fe^2+^ transporter in yeast and transgenic *Arabidopsis*. *ZmIRT1* overexpression plants contain increased amounts of Zn and Fe in roots and seeds (Fig 4A and 4E), as well as elevated Zn levels in seedlings (Figs 5 and 6). In addition, *ZmZIP3*-overexpressing transgenic plants accumulated more Zn in roots. These results indicated that ZmIRT1 and ZmZIP3 are functional ion transporters. Moreover, we identified putative *FRO* and *AHA* genes in the maize genome (data not shown) using the reported AtFRO and AtAHA (H^+^-ATPase) as TBLASTN queries, and suggested that the ferric reducing and rhizosphere acidification capacities may be retained through evolution. Thus, further investigations are required to determine whether maize use strategy I as a complementary Fe uptake mechanism, and determine why it has been maintained during evolution.

It was previously reported that although *AtIRT1* was expressed constitutively in *35S-IRT1* transgenic plants, the protein was present only in iron-limited roots. This result suggested that the accumulation of *IRT1* is influenced by post-transcriptional regulation [58]. It was also reported that post-transcriptional regulation did not play a significant role in *OsIRT1* overexpression rice, as both *OsIRT1* and metals were accumulated under metal-sufficient conditions and the transgenic plants showed visible morphological changes when grown under standard conditions [25]. In this study, the *ZmIRT1*-overexpressing *Arabidopsis* exhibited increased levels of zinc and iron in different tissues, and the transgenic plants showed an enhanced resistance against excess-iron and zinc. These results indicated that *ZmIRT1* might escape post-transcriptional control because ZmIRT1 is highly homologous with OsIRT1 (73.04% identity) and they share low sequence similarity with AtIRT1 (50.92% identity for ZmIRT1 and 48.93% identity for OsIRT1, respectively).

## Conclusions

In conclusion, we generated transgenic *Arabidopsis* plants overexpressing *ZmIRT1* or *ZmZIP3* to explore the function of these genes in metal uptake, translocation, and homeostasis. As expected, the Fe and Zn contents were enhanced in roots and mature seeds of *ZmIRT1* overexpression plants, while the Zn level was increased in roots of *ZmZIP3*-overexpressing lines. Likewise, zinc staining revealed that the Zn ions accumulated in the seedlings of both *ZmIRT1* and *ZmZIP3* transgenic plants. These results indicate that ZmIRT1 and ZmZIP3 are functional metal transporters, while they have different selectivity towards various metal ions. These results may be applied in molecular breeding and biofortification of maize with micro-essential metal nutrients.

## Supporting Information

S1 Fig Subcellular localization of ZmIRT1 and ZmZIP3 in maize mesophyll protoplasts. Full-length cDNA without stop codon of the *ZmIRT1* and *ZmZIP3* genes were cloned into the pRTL2GFP vector and the resulting construct was transiently transformed into maize mesophyll protoplasts by the PEG method. The GFP signal is shown in green and the fluorescence of ER marker is indicated in red. The images were obtained by a confocal microscope. The scale bar represents 10 μm.
(TIF)

S1 Table Primers used for vector construction and quantitative RT-PCR analysis. (DOCX)
