# Supplementary figures and images for: Overexpression of ZmIRT1 and ZmZIP3 Enhances Iron and Zinc Accumulation in Transgenic Arabidopsis
Source: PLoS One. 2015 Aug 28;10(8):e0136647. doi: 10.1371/journal.pone.0136647 (PMC4552944; doi:10.1371/journal.pone.0136647)

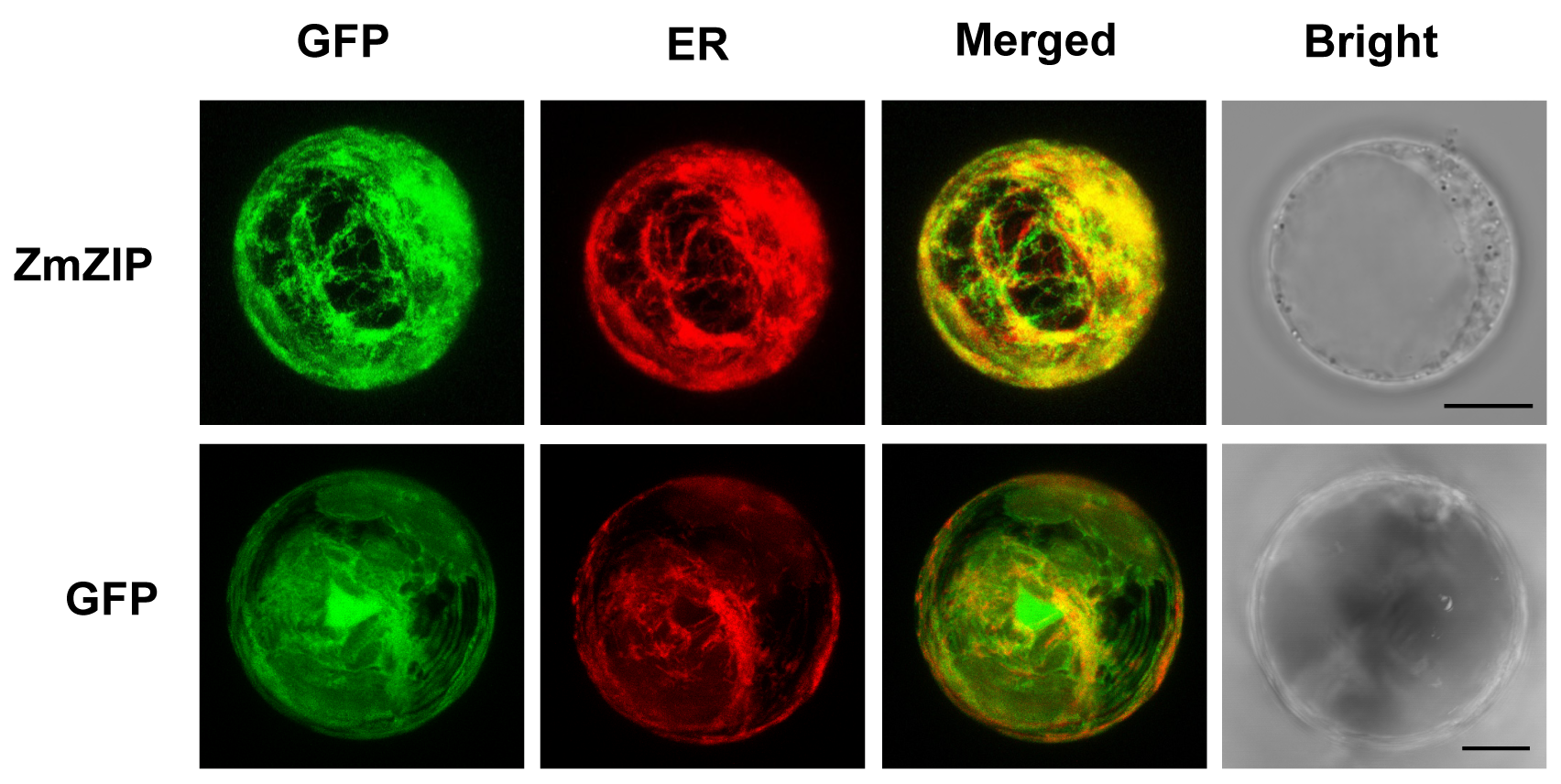

Supplement: S1 Fig — Full-length cDNA without stop codon of the ZmIRT1 and ZmZIP3 genes were cloned into the pRTL2GFP vector and the resulting construct was transiently transformed into maize mesophyll protoplasts by the PEG method. The GFP signal is shown in green and the fluorescence of ER marker is indicated in red. The images were obtained by a confocal microscope. The scale bar represents 10 μm. (TIF) [file pone.0136647.s001.tif]
