# Supplementary material for: Overexpression of ZmIRT1 and ZmZIP3 Enhances Iron and Zinc Accumulation in Transgenic Arabidopsis
Source: PLoS One. 2015 Aug 28;10(8):e0136647. doi: 10.1371/journal.pone.0136647 (PMC4552944; doi:10.1371/journal.pone.0136647)
Supplement: S1 Table — (DOCX) [file pone.0136647.s002.docx]

| **Primer names** | **Primer sequences** | **Enzyme (underlined)** |
| --- | --- | --- |
| IRT1oxF | 5' GCGGCCGCTCTAGAATGTCTTGGCGGCGAACAC 3' | *XbaI* |
| IRT1oxR | 5' GCGGCCGCTACGTATCACGCCCACTTGGCCATGATG 3' | *SnaBI* |
| ZIP3oxF | 5' CCCGGGATGGGAGCTGTGAAGCATACATTG 3' | *SmaI* |
| ZIP3oxR | 5' GGTACCCTATGCCCATATAGCAAGCATGGAC 3' | *KpnI* |
| ZmIRT1GF | 5' CTCGAGATGTCTTGGCGGCGAAACC 3' | *XhoI* |
| ZmIRT1GR | 5' TCTAGACGCCCACTTGGCCATGATG 3' | *XbaI* |
| ZmZIP3GF | 5' GAATTCATGGGAGCTGTGAAGCATAC 3' | *EcoRI* |
| ZmZIP3GR | 5' TCTAGATGCCCATATAGCAAGCATGGACAT 3' | *XbaI* |
| AtUBP6F | 5' GAAAGTGGATTACCCGCTG 3' |  |
| AtUBP6R | 5' CTCTAAGTTTCTGGCGAGGAG 3' |  |
| AtNAS1F | 5' TGTGGTGAAGCCGAAGGTTA 3' |  |
| AtNAS1R | 5' GGACCCGAACCCACGAAG 3' |  |
| AtNAS2F | 5' GCTTACCAACGGACACGAAC 3' |  |
| AtNAS2R | 5' GTTGTTGTAATAGGGGAAGATGTG 3' |  |
| AtIRT1F | 5' GAATGTGGAAGCGAGTCAGC 3' |  |
| AtIRT1R | 5' TGAAGATGTTTCCGTCTGGTT 3' |  |
| AtFRO2F | 5' CTTGGTCATCTCCGTGAGC 3' |  |
| AtFRO2R | 5' AAGATGTTGGAGATGGACGG 3' |  |
| AtFITF | 5' ATGGGACCCGTAAGACGAAGAC 3' |  |
| AtFITR | 5' ATCGGATTTGAGTTTCTTCGCT 3' |  |
| AtNRAMP2F | 5' TTGTGGCTACTAATGTGGGCA 3' |  |
| AtNRAMP2R | 5' CGCCATAGACCAAAGCACATA 3' |  |
| AtNRAMP3F | 5' TGCCTCTTTGGGCTGGTG 3' |  |
| AtNRAMP3R | 5' CAAACATCCAAGCGAATGAGACT 3' |  |
| AtBHLH100F | 5' AAGTCAGAGGAAGGGGTTACA 3' |  |
| AtBHLH100R | 5' GATGCATAGAGTAAAAGAGTCGCT 3' |  |
| AtYSL1F | 5' ATGACATTTGTGGGAGCAGG 3' |  |
| AtYSL1R | 5' CGTCGCCGAGGATTAGAGC 3' |  |
| AtYSL2F | 5' TACAACTATGGTAAAGCGGCG 3' |  |
| AtYSL2R | 5' GGTTTGCGTAAGATGTCCCGT 3' |  |
| AtZIP2F | 5' CTTACTTTTACCGATGGAACGA 3' |  |
| AtZIP2R | 5' TACAAACGCAACCGCCA 3' |  |
| AtZIP4F | 5' GCCATTGCCTCCATTCTACTA 3' |  |
| AtZIP4R | 5' GGGAAACTTAGACCACGGAAA 3' |  |
